# A Hybrid Energy-Efficient, Area-Efficient, Low-Complexity Switching Scheme in SAR ADC for Biosensor Applications

**DOI:** 10.3390/mi15010060

**Published:** 2023-12-27

**Authors:** Yunfeng Hu, Chaoyi Chen, Qingming Huang, Lexing Hu, Bin Tang, Mengsi Hu, Bingbing Yuan, Zhaohui Wu, Bin Li

**Affiliations:** 1 Zhongshan Institute, University of Electronic Science and Technology of China, Zhongshan 528402, China; 2 School of Microelectronics, South China University of Technology, Guangzhou 510640, China

**Keywords:** SAR, low complexity, energy efficiency, area efficiency, capacitor splitting, monotonic

## Abstract

A hybrid energy-efficient, area-efficient, low-complexity switching scheme in SAR ADC for biosensor applications is proposed. This scheme is a combination of the monotonic technique, the MSB capacitor-splitting technique, and a new switching method. The MSB capacitor-splitting technique, as well as the reference voltage *V*_aq_ allow for more options for reference voltage conversion, resulting in higher area savings and higher energy efficiency. In a capacitor array, the circuit performs unilateral switching during all comparisons except for the second and last two comparisons, reducing the difficulty in designing the drive circuit. The proposed switching scheme saves 98.4% of the switching energy and reduces the number of unit capacitors by 87.5% compared to a conventional scheme. Furthermore, the SAR ADC employs low-noise and low-power dynamic comparators utilizing multi-clock control, low-sampling error-sampling switches based on the bootstrap technique, and dynamic SAR logic. The simulation results demonstrated that the proposed SAR ADC achieves 61.51 dB SNDR, 79.21 dB SFDR and consumes 0.278 μW of power in a 180 nm process with a 1 V power supply, a full swing input signal frequency of 23.33 kHz, and a sampling rate of 100 kS/s.

## 1. Introduction

**Figure 1:**
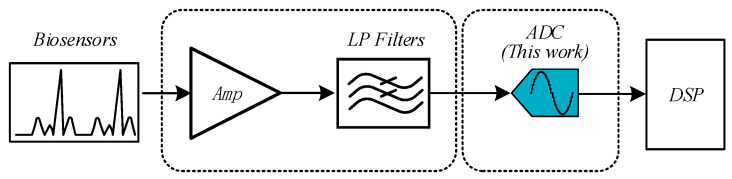
Block diagram of biomedical implantable device.

The development of a variety of portable biometric monitoring devices is currently being promoted, a trend that requires tight size and power constraints for electrical signaling systems at the backend of biosensors. As a result, low-power implantable biosensors or signal processing systems have been extensively developed to extract and analyze signals. [1,2]. Figure 1 shows the basic processing units in a biomedical implantable device. Biosensors convert a variety of vital signs into usable electrical signals, which are pre-processed by amplifiers and filters, and then converted into digital signals by ADCs, which are processed by DSPs for subsequent use. In order to obtain high-quality biometric signals (digital signals), low-noise amplifiers and medium-high precision ADCs are required [3].

In recent years, SAR ADCs have gained popularity as low-power ADCs [4,5,6]. Simulations have shown that the DAC capacitor array usually accounts for a large part of the overall power consumption of SAR ADCs [7,8]. In order to cope with this energy structure, a number of energy-saving schemes have recently been proposed, including ERMS [9], Tri-level [10], VMS [11] and Hybrid [12]. That of the C-2C common-mode voltage structure DAC can be reduced by 90.61% [9], that of the tri-level structure DAC can be reduced by 97.66% [10], that of the common-mode voltage monotonic (VMS) structure DAC can be reduced by 96.89% [11], and that of the hybrid capacitor structure DAC be reduced by 98.83% [12]. In addition to these, schemes have also been presented by Wang et al. [13], Li et al. [14], Sanyal et al. [15], Wu et al. [16], MAS [17], and Huang et al. [18], achieving energy savings of 98.1%, 98.0%, 98.4%, 98.43%, 98.45%, and 99.5%, respectively. ERMS [9] uses the C-2C dummy capacitor, which reduces the overall area and the number of switches, improving energy efficiency. Tri-level [10] introduces an additional reference voltage, *V*_cm_, and uses three reference voltage switching in the switching scheme, which greatly improves energy efficiency. VMS [11] also uses *V*_cm_ as extra reference voltage, achieving higher energy efficiency and a lower common-mode voltage variation. Hybrid [12] is a perfect application of *V*_cm_, which saves a lot of overall energy compared to the above scheme using *V*_cm_, but also brings a large common-mode voltage variation. Wang et al. [13] uses a bridge switch and four reference voltages, which results in higher efficiency and a lower common-mode voltage variation, but also increases the logic complexity of the circuit.

The advantages of the switching scheme proposed in this paper are not only in the high energy savings, but also in the high area savings. Similar energy savings schemes have been presented by Li et al. [14] and Huang et al. [18]; however, their designs also have their own characteristics. In the scheme by Li et al. [14], C/2 is used as the unit capacitor, a bridge switch is applied for in the second conversion step, and all capacitors are connected to *V*_cm_, which is highly dependent on the *V*_cm_. Furthermore, all capacitors are switched by three reference voltages, which increases the complexity of the logic circuit. The scheme by Huang et al. [18] has a higher energy efficiency, and at the same time, it suffers from higher common-mode voltage variation as *V*_aq_ appears throughout the comparison, raising the dependence on *V*_aq_. Wu et al. [16] and MAS [17] use a floating technique that replaces the additional reference voltage, achieves higher energy efficiency, lower common-mode voltage variation, and reduces the dependence on the additional reference voltage. However, the area savings of these schemes are relatively limited, and the complexity of the drive circuit design is also increased. This paper proposes a switching scheme based on *V*_aq_ (1/4*V*_ref_) with low complexity, which not only achieves a relatively high energy-efficiency ratio, but also has a smaller area compared with the above schemes.

## 2. Design of the Proposed SAR ADC

**Figure 2:**
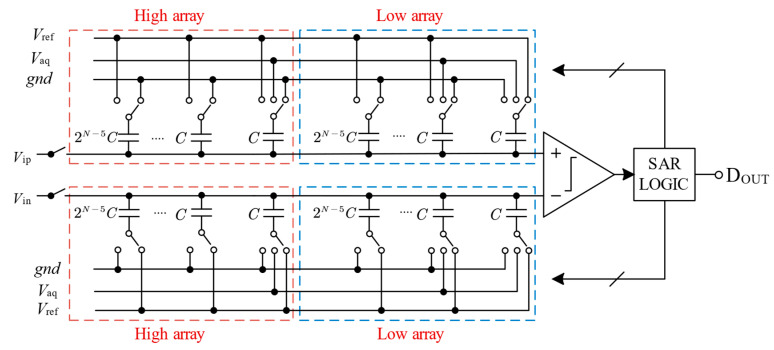
Proposed 10-bit SAR ADC architecture.

The structure of the N-bit SAR ADC is demonstrated in Figure 2. To suppress supply voltage noise and achieve good common-mode noise rejection, we have adopted a fully differential architecture [19]. The fundamental blocks of an SAR ADC are the sample-and-hold circuit, the comparator, the capacitive DAC, and successive approximation registers.

The DAC array consists of a positive array and a negative array, with a high array and a low array in each array. In the high array, only the dummy capacitor is a three-switch, while in the low array, the minimum bit capacitor and the dummy capacitor are both three switches. A reference voltage, *V*_aq_, is used for the last two comparisons, and the use of *V*_aq_ reduces the total amount of unit capacitors by 87.5% compared to the conventional scheme. In addition, only 6-unit capacitors apply for *V*_aq_ as the reference voltage in the last two steps, and the remaining capacitors have only two reference voltages, which reduces the complexity of the circuit design.

### 2.1. The Analysis of Switching Scheme

**Figure 3:**
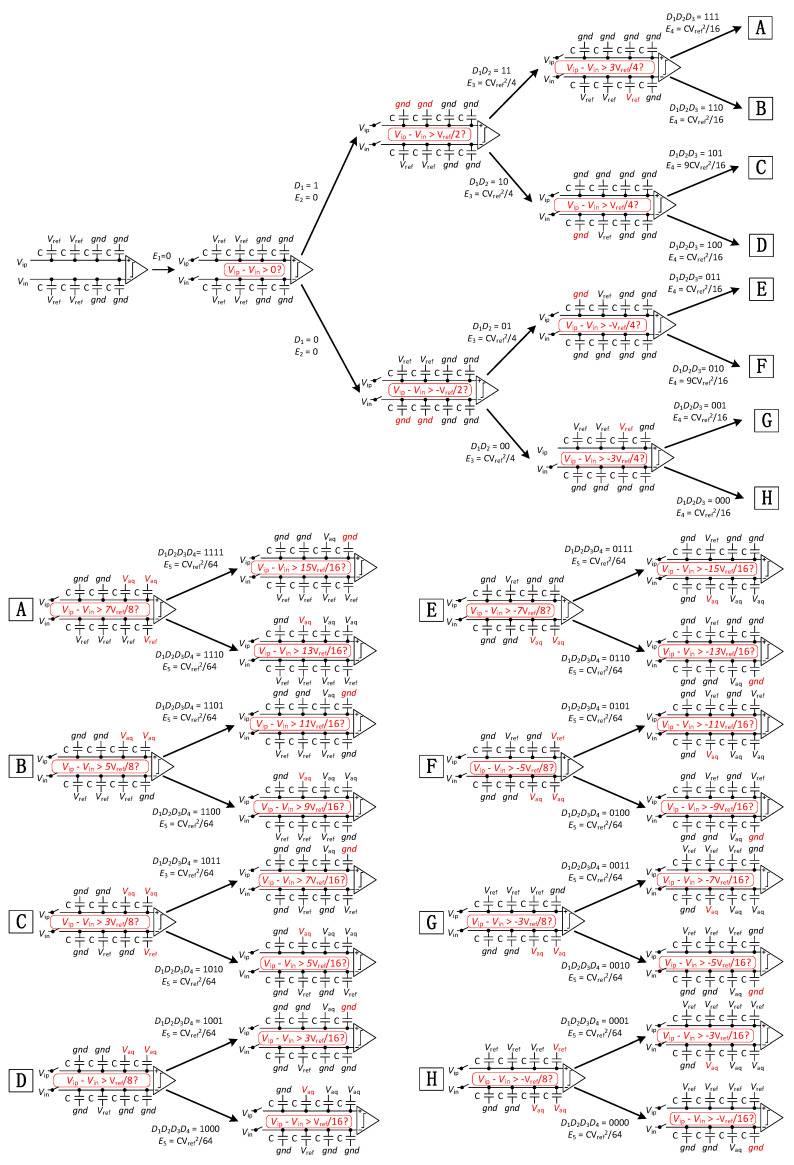
Switching scheme of 5-bit SAR DAC.

To explain the principle of the proposed switching scheme, the 5-bit SAR ADC conversion diagram is shown in Figure 3. The whole switching process can be divided into five phases:

Phase 1: the sampling switch samples the input signal on the top plate of all capacitors. The bottom plates of the high array are connected to *V*_ref_, The bottom plates of the low array are connected to *gnd*. After sampling, the sampling switch is turned off. Then, the comparator performs the first comparison and outputs the first comparison result, *D*_1_, without consuming any switching energy;

Phase 2: based on the previous output of the comparator, the bottom plates with all capacitors on the higher side of the voltage switches for the high array from *V*_ref_ to *gnd*, while the other arrays remain unchanged. As a result, the voltage on the higher voltage side decreases to *V*_ref_/2, while the voltage on the low voltage side does not change. The comparator then performs a second comparison and outputs the results of the second comparison, *D*_2_, without consuming any switching energy;

Phase 3: depending on the previous output of the comparator and the comparison of the results, *D*_1_, the 3rd to the (*N* − 2)th comparison is executed. When *D*_1_*D_i_* is 11 or 00, the low array corresponding capacitor (2*^N^*^−*i*−2^C) bottom plate on the lower voltage side is switched from *gnd* to *V*_ref_, while the other arrays remain unchanged. When *D*_1_*D_i_* is 10 or 01, the high array corresponding capacitor (2*^N^*^−*i*−2^C) bottom plate on the higher voltage side is switched from *V*_ref_ to *gnd*, while the other arrays remain unchanged.

The ADC repeats the process until the completion of the (*N* − 1)th comparison is complete. The process is also called a loop bit. At the loop bit of the conversion process, the voltage on one side remains unchanged. The capacitor array switching energy for each comparison from the 3rd to the (*N* − 2)th comparison is:

(1) $$E\left(i\right)=\left\{\begin{array}{c} -\frac{\left[\left(1-2D\left(i-1\right)\right)\right]}{2^{i-1}}\cdot \left[\begin{array}{c} \sum_{x=5}^{i+2}2^{N-x}\cdot \left(1-D_{1}-D_{x-3}\right)+\sum_{j=i+3}^{N}2^{N-j}\cdot \left(1-2D_{1}\right)+\left(1-2D_{1}\right)+\\ \sum_{m=5}^{i+1}\begin{array}{c} 2^{N-m}\cdot \left(1-D_{1}-D_{m-3}\right) \end{array}+2^{N-i-2}\cdot \left(1-D_{1}-D_{i-1}\right) \end{array}\right]\\ +2^{N-i-2}\cdot \left(1-D_{1}-D_{i-1}+2D_{1}D_{i-1}\right) \end{array}\right\}C{V_{ref}}^{2}$$

Phase 4: Depending on the (*N* − 2)th comparison and comparison of results *D*_1_, the (*N* − 1)th comparison is performed. When *D*_1_*D_n_*_−2_ is 11 or 00, the low array corresponding capacitor (C, C) bottom plate on the higher voltage side is switched from *gnd* to *V*_aq_, the low array corresponding capacitor (C) bottom plate on the lower voltage side is switched from *gnd* to *V*_ref_, and the other arrays remain unchanged. When *D*_1_*D_n_*_−2_ is 10 or 01, the low array corresponding capacitor (C, C) bottom plate on the lower voltage side is switched from *gnd* to *V*_aq_, while the other arrays remain unchanged. The capacitor array switching energy for the (*N* − 1)th comparison is:

(2) $$E\left(n-1\right)=\left\{\begin{array}{c} -\frac{1}{2^{n-2}}\left[\begin{array}{c} 4\cdot (1-D_{1}-D_{n-2}+2D_{1}D_{n-2})+\frac{1}{2}\\ +\sum_{i=5}^{n}2^{N-i}\cdot 2\cdot 2(1-D_{1}-D_{j-3}-D_{n-2}+D_{j-3}D_{n-2}+D_{1}D_{n-2}+D_{1}D_{j-3}) \end{array}\right]\\ +\frac{1}{8}+\left(1-D_{1}-D_{n-2}+2D_{1}\cdot D_{n-2}\right) \end{array}\right\}C{V_{ref}}^{2}$$

Phase 5: the *N*th comparison is performed based on the (*N* − 1)th output of the comparator. When *D*_1_*D_n_*_−1_ is 11 or 00, the high array corresponding capacitor (C) bottom plate on the higher voltage side is switched from *V*_aq_ to *gnd*, while the other arrays remain unchanged. When *D*_1_*D_n_*_−1_ is 10 or 01, the low array corresponding capacitor (C) bottom plate on the lower voltage side is switched from *gnd* to *V*_aq_, and the other arrays remain unchanged. The capacitor array switching energy for the *N*th comparison is:

(3) $$E\left(n\right)=\left[\left(-4D_{n-1}+8D_{1}D_{n-1}-4D_{1}+1\right)\frac{1}{2^{n+1}}+\frac{1}{16}\left(D_{1}+D_{n-1}-2D_{1}D_{n-1}\right)\right]C{V_{ref}}^{2}$$

For the N-bit resolution, the average switching energy of the capacitor array switching energy is:

(4) $$E_{average}=\left(2^{N-2i-1}+\frac{21}{32}-\frac{5}{2^{n-1}}-\sum_{i=5}^{N}2^{2-i}\right)C{V_{ref}}^{2}$$

**Figure 4:**
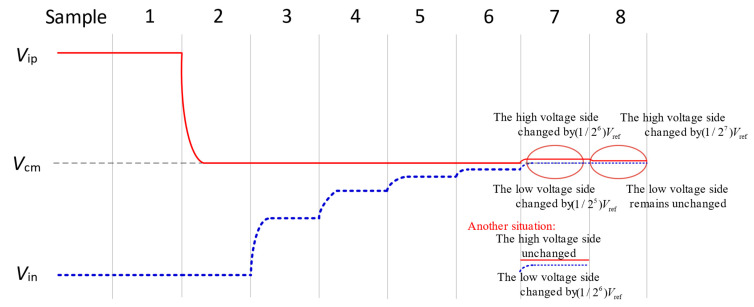
Waveform of the proposed switching scheme.

The 8-bit successive approximation voltage waveforms of the proposed switching scheme are illustrated in Figure 4. The maximum common-mode variation occurs at the second comparison, with a value of *V*_ref_/4. At the loop bit of the conversion process, the scheme performs unilateral switching, its voltage on one side remains constant. The last two voltage changes are labelled in detail in the figure; in the second-to-last voltage conversion, the positive array and negative array voltages change at the same time, the voltage of the positive array is increased by *V*_ref_/2*^n^*^−3^, while the voltage of the negative array is increased by *V*_ref_/2*^n^*^−2^, but the total change in the voltage is *V*_ref_/2*^n^*^−2^; and the last voltage change for the positive array side changes to *V*_ref_/2*^n^*^−1^, all in line with the successive approximation principle. This feature makes it possible to focus only on one side of the drive logic design in most cases, reducing the complexity of the drive circuit design.

### 2.2. DAC Driver Circuit Design

**Figure 5:**
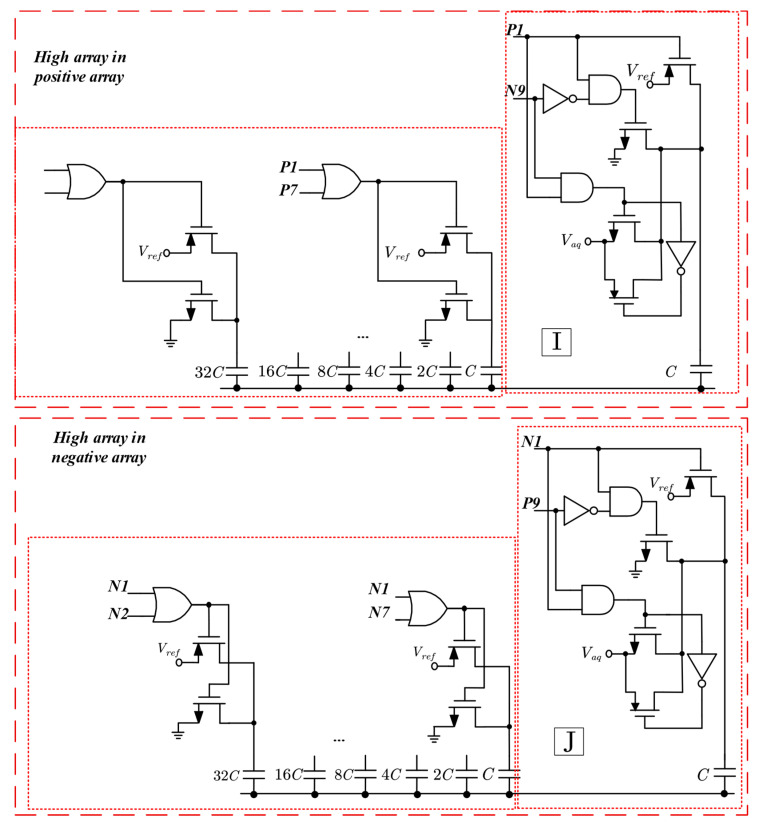
Drive circuit of high array of positive array and negative array.

**Figure 6:**
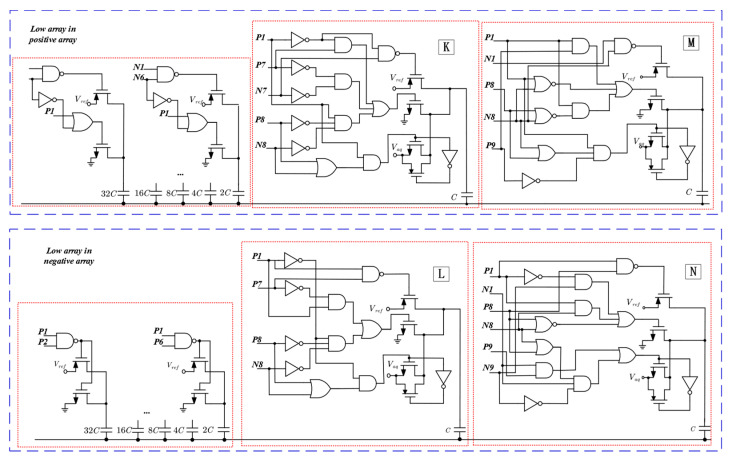
Drive circuit of low array of positive array, and negative array.

The drive circuits of the switching scheme proposed in this paper are shown in Figure 5 and Figure 6. Figure 5 characterizes the capacitor drive logic for the high array in the positive and negative array, and Figure 6 represents the capacitor drive logic for the high array in the positive and negative array. The drive logic consists of a combination of various logic gates, and the drive logic signal input is derived from the output of the SAR logic, the input signals P and D are the same signals, and the N signal is inverted from the D signal, but the initial values of D and N are both 0. Since a third reference voltage, *V*_aq_ (1/4*V*_ref_), is used, the reference voltage drive transistor uses a logic gate structure to eliminate the threshold loss and reduce the on-resistance.

The truth table of the driver circuits I and J is illustrated in Table 1. In the switching logic, the control of the lowest bit capacitor in the high array requires six signals. However, through calculation and simplification, this can be reduced to only two signals. For instance, in the high array, the connection of the top plate of the capacitor to *V*_ref_ occurs only when P1 is 0. Hence, *V*_ref_ can be controlled using only P1. On the other hand, the top plate of the capacitor is linked to *gnd* only when P1 is 1 and N9 is 0, so *gnd* can be controlled by using P1, N9, and their combination, $P1\cdot \bar{N9}$. The control logic for *V*_aq_ follows a similar pattern, expressed as P1⋅N9.

The truth table of the driver circuit K and L is depicted in Table 2. Similar to Table 1, this capacitor also requires control by six signals. However, the control logic for this capacitor is slightly more complex. By simplifying the truth table in a similar manner to that presented above, the control logic for *gnd* in the low array can be expressed as $P1\cdot \bar{P7}+\bar{P1}\cdot \bar{P8}\cdot \bar{N8}$, while the control logic for *V*_ref_ in the low array can be obtained as $\bar{P1\cdot P7}$, and the control logic for *V*_aq_ in the low array can be obtained as $\bar{P1}\cdot (P8+N8)$.

The truth table of the driver circuit M and N is depicted in Table 3. The control logic for gnd in the high array is expressed as $\bar{P1+N8}+P1\cdot P9\cdot +P1\cdot \bar{\left(P8+N8\right)}$, while the control logic for V_ref_ in the high array can be obtained as $\bar{N1\cdot N8}$, and the control logic for V_aq_ in the high array is represented as $P1\cdot \bar{\left(P8+N8\right)}\cdot \bar{P9}$.

### 2.3. Switching Energy Analysis and Comparison

**Figure 7:**
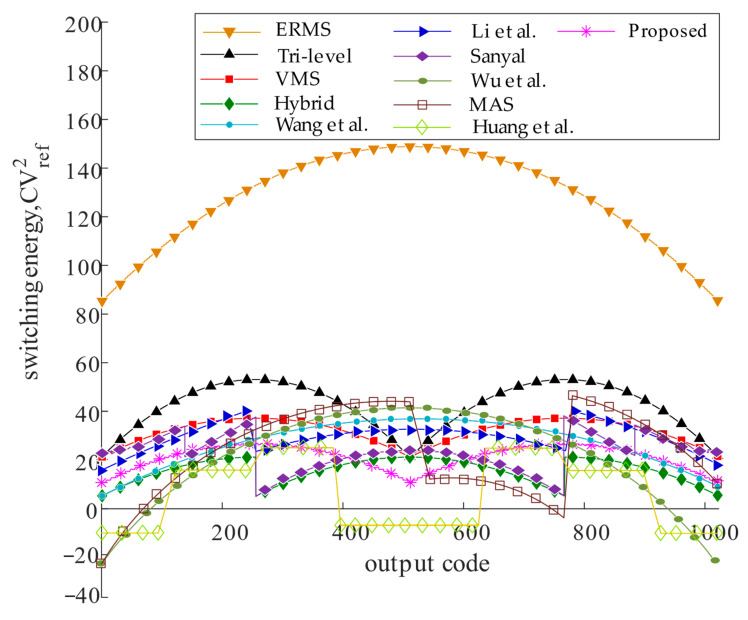
Switching energy against output codes [9,10,11,12,13,14,15,16,17,18].

Behavioral simulation of the switching scheme for the 10-bit SAR ADC was implemented in MATLAB. The switching energy of the different schemes under each code is shown in Figure 7. Compared with most schemes, this scheme has no disadvantages in terms of energy efficiency. The average switching energy of the proposed 10-bit SAR ADC capacitive switching scheme is 21.24 *CV*^2^_ref_. Compared to the conventional switching scheme, the switching energy is reduced by 98.4%. Table 4 and Table 5 show the performance characteristics of different switching schemes for the 10-bit SAR ADC. Compared to the switching scheme [9,10,11,12,13,14,15,16,17] in the table, ERMS [9] uses a C-2C dummy capacitor and tri-level [10], Sanyal [15], VMS [11] introduces an additional reference voltage, *V*_cm_, Wang et al. [13] uses a bridge switch and four reference voltages, Wu et al. [16] uses floating technique, which replaces the additional reference voltage. The scheme in this study has a lower average switching energy, achieving 98.44% energy savings. Moreover, MAS [17] uses floating technique that achieves higher energy efficiency; however, the scheme presented in this study has fewer total unit capacitors, achieving an 87.5% area reduction with excellent energy savings.

The scheme presented by Huang et al. [18] is similar to that presented in this paper in terms of its DAC circuit structure; both use the MSB capacitor-splitting technique and additional reference *V*_aq_, but the difference between the two is that *V*_aq_ is only used in the last two reference voltage switches for the proposed scheme. Huang et al. [18] use *V*_aq_ throughout the comparison, and its scheme does bring higher energy efficiency and effectively reduces the capacitor area, resulting in a larger common-mode voltage variation, which improves the dependence of the overall circuit on *V*_aq_, and increases the complexity in the design of the drive circuits. Compared to Huang’s scheme, the advantages of the scheme presented in this paper include a lower common-mode voltage variation and lower dependence on *V*_aq_.

In addition, the design is characterized by low complexity, which is the result of a comparison with most of the energy-efficient schemes in Table 5. This is because the design only needs to change the reference of one corresponding capacitor during the loop bit, and no additional reference voltage is required. *V*_aq_ is only used in the last two comparisons, which also reduces the dependence on *V*_aq_ and weakens the logical complexity of the switching scheme. Low-complexity features in the drive circuit are more specific, the drive circuit has a small number of logic gates; the one-bit control has only three logic gates; the drive circuit has fewer control signals; and the number of switches in the circuit is low.

The capacitor mismatch, which occurs often in the CMOS process, leads to integral nonlinearity (INL) and differential nonlinearity (DNL) of the DAC and deteriorates the accuracy of the A/D conversion.

### 2.4. Analysis of Noise

Lowering the supply voltage is an efficient technique to reduce both the switching and leakage power consumption. However, for analog circuits operating with low supply voltages, noise has a greater impact on ADC performance [20]. The novel switching scheme proposed here utilizes a capacitor-splitting structure to save the number of capacitors, reducing the area by 75% compared to the conventional switching scheme. The reduced number of capacitors will have an effect on the thermal noise of the ADC, which, in SAR ADC circuits, can randomly interfere with the voltage and current in the sampling circuits, thus affecting the accuracy of the ADC. The usual form of thermal noise is kT/C [21,22]. Ref. [23] presents a generalized statistical model for calculating the noise of a SAR ADC. The total input reference noise $\sigma_{total}^{2}$ of the proposed SAR ADC is calculated by Equation (5):

(5) $$\sigma_{total}^{2}=\sigma_{thermal}^{2}+\sigma_{comparator}^{2}+\sigma_{quantization}^{2}$$

where $\sigma_{thermal}^{2}$ is the thermal noise from capacitor arrays, $\sigma_{comparator}^{2}$ is the input-referred noise from the comparator and $\sigma_{quantization}^{2}$ is the quantization noise.

Considering the mismatch, signal build-up time, and noise, a capacitor of 17.2 fF is selected as the unit capacitance value C_u_ and the on-resistance of the most significant bit (MSB) capacitive control switch, denoted by R_on_, is 7.3 kΩ, which satisfies the RC time constant and thermal noise requirements. The low and high arrays in the capacitor array are incremented in binary capacitance order, respectively. The value of σ_thermal^2 can be obtained as follows:

(6) $$\sigma_{thermal}=\sqrt{\frac{kT}{C_{total}}}$$

where *k* is the Boltzmann constant, *T* is the Kelvin temperature.

The number of capacitors for the proposed switching scheme is only 256. According to Equation (6), the value of $\sigma_{thermal}$ for the proposed method is 30.66 μV.

To improve energy efficiency and reduce overall power consumption, the unit capacitance in the DAC should be as small as possible. However, mismatch and thermal noise limit the size of the unit capacitance. Therefore, the selection of the unit capacitance should weigh the switching energy, speed, linearity, and thermal noise. Capacitor values should be designed to meet the thermal noise, which is usually less than the quantization noise ($\sigma_{quantization}^{2}$). The quantization noise power can be expressed as

(7) $$\sigma_{quantization}^{2}=\frac{{\left(V_{ref}/2^{N}\right)}^{2}}{12}$$

where *V*_ref_ represents the peak-to-peak voltage swing of the input signal. In a 10-bit SAR ADC with a reference voltage of 1 V, the quantization error can be calculated as 0.282 mV, according to the above equation. The relationship between the sampling noise and the quantization noise can be expressed as

(8) $$\sigma_{thermal}^{2}\leq \sigma_{quantization}^{2}$$

Through (6)–(8), the total capacitance can be obtained. The total capacitance needs to be larger than 52.1 fF to meet the thermal noise requirement at a 1 V peak-to-peak differential signal swing in a 10-bit SAR ADC. The results show that the designed unit capacitance value meets the requirements in terms of thermal noise. In a typical design, $\sigma_{comparator}^{2}\;\;$ dominates by over a factor of 100, or over $\sigma_{thermal}^{2}$. Therefore, the $\sigma_{comparator}$ of the proposed SAR ADC is 0.89 mV. As a result, the thermal noise has a limited effect on the total noise since it is only a small part of the total noise.

### 2.5. Linearity

#### 2.5.1. Effect of Capacitor Mismatch on Linearity

For capacitor array DACs, the capacitor mismatch phenomenon, which is widespread in CMOS processes, dominates the linearity of the ADC. While keeping the value of C_u_ as small as possible, it is more important to consider the effects of capacitor mismatch than thermal noise [21]. The capacitor mismatch which widely exists in the CMOS process leads to integral nonlinearity (INL) and differential nonlinearity (DNL) of DAC and deteriorates the accuracy of A/D conversion.

Considering the capacitor mismatch, and assuming that the unit capacitor obeys a Gaussian distribution, the standard deviation of the capacitance model is σ_u_.

**Figure 8:**
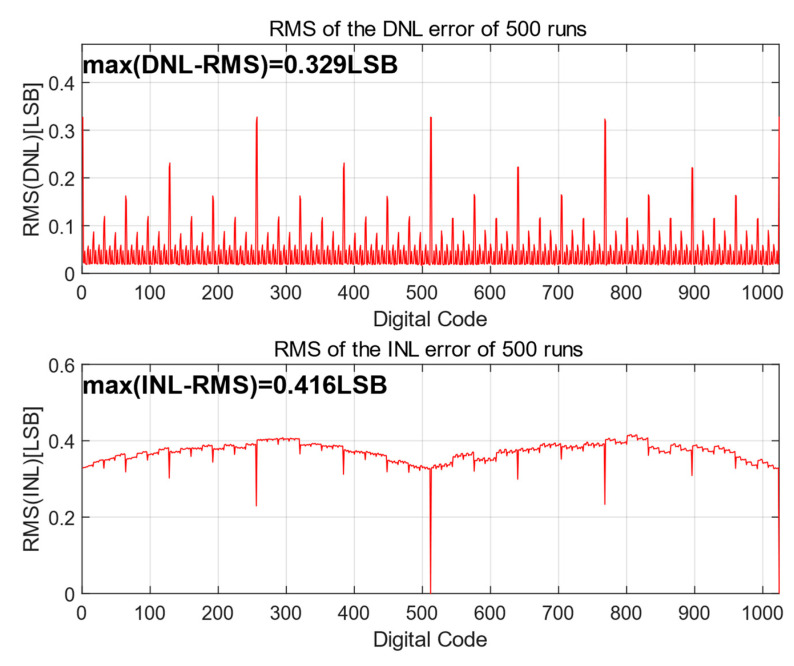
The simulated RMS results of DNL and INL.

To simulate the influence of the capacitors’ mismatch on linearity, Figure 8 presents the 500-run Monte Carlo simulation results of the proposed switching scheme with C_u_ of σ_u_/C = 1% [22]. The root-mean-square (RMS) DNL and the RMS INL of the proposed switching scheme are only 0.328 LSB and 0.414 LSB. From the simulation results, the proposed switching scheme has good performance in terms of linearity.

#### 2.5.2. Effect of Mismatch between *V*_aq_ on Linearity

**Figure 9:**
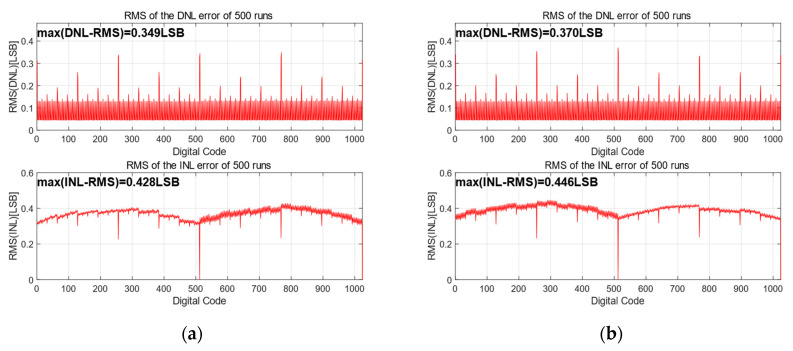
The simulated RMS results for increasing *V*_aq_ mismatch: (**a**) *V*_aq_ = *V*_ref_/4 + 1%*V*_ref_.; and (**b**) *V*_aq_ = *V*_ref_/4 − 1%*V*_ref_.

In order to simulate the effect of *V*_aq_ accuracy on the proposed switching scheme, the mismatch value of *V*_aq_ between *V*_ref_ and *V*_aq_ is set to 1% (*V*_aq_ = *V*_ref_/2 ± 1%*V*_ref_) from its ideal value of *V*_ref_ and added in the simulation on the basis of the simulated capacitor mismatch. A total of 500 Monte Carlo runs were performed with the proposed 10-bit SAR ADC. Figure 9 illustrates the simulation results between the proposed switching schemes. For the proposed switching scheme, the maximum DNL and INL RMS are 0.357 LSB and 0.432 LSB, respectively. After considering the mismatch between *V*_ref_ and *V*_aq_, the DNL and INL do not change significantly compared with Figure 7 and satisfy the linearity requirement, indicating that the accuracy of *V*_aq_ in the proposed switching scheme has less impact on the overall accuracy of the DAC.

### 2.6. Circuit Implementation

The proposed SAR ADC includes two gate-voltage bootstrap switches as sampling switches, a dynamic comparator for voltage comparison, a set of DAC capacitor arrays and a dynamic SAR controller for the DAC network. The following sections describe the circuit implementation and design considerations for each key module circuit.

#### 2.6.1. Bootstrapped S/H Switch

**Figure 10:**
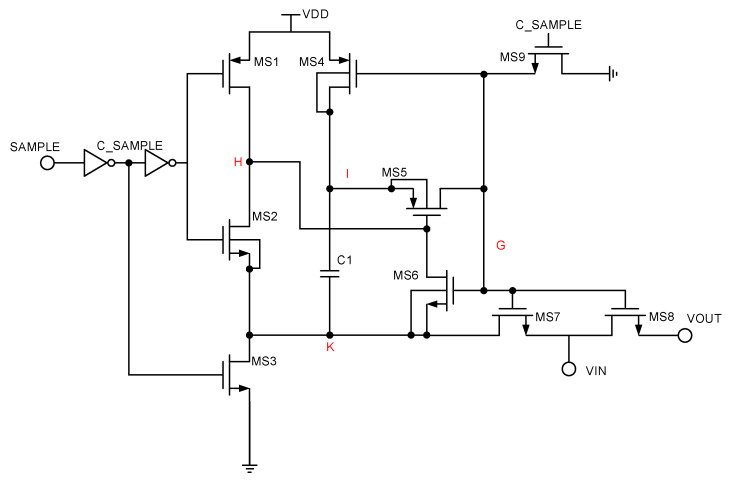
Bootstrapped sampling switch.

**Figure 11:**
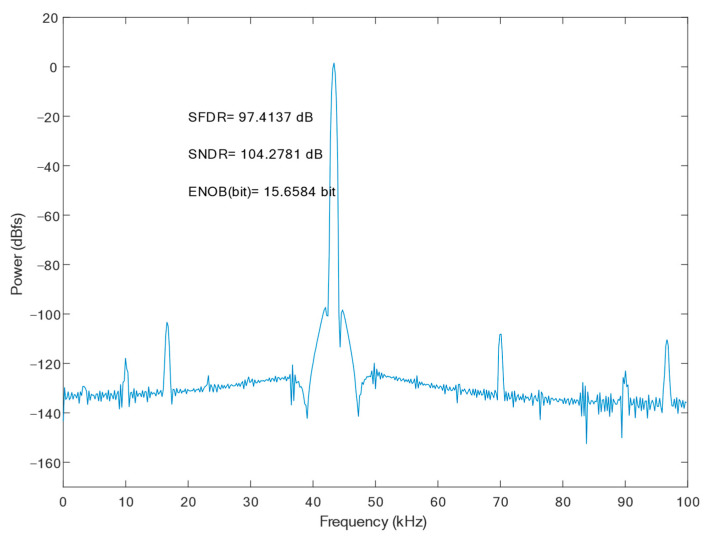
FFT of Bootstrapped sampling switch.

**Figure 12:**
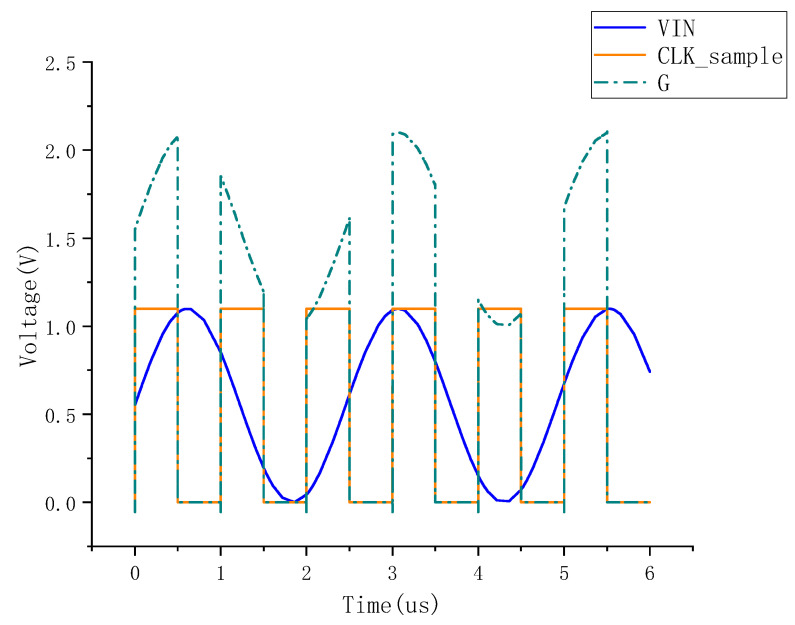
Bootstrapped sampling switch transient simulation.

In order to enhance the performance of sampling switches and reduce the sampling error, bootstrap circuits are often used in sampling switches. The bootstrap switch improves the on-resistance stability of the sampling switch [24,25,26]. The bootstrap process is divided into two phases, as shown in Figure 10. During the turn-on phase, charge conservation is utilized for the gate capacitance of the MS8, the C1, and other parasitic capacitances, resulting in the voltage at the G point being VDD + VIN and the output at the VOUT side being Vin, so that the on-resistance of the sample switch MS8 remains constant. Figure 11 shows the spectral analysis results of the bootstrap sampling switch. Figure 12 shows the transient simulation of the bootstrap sampling switch.

#### 2.6.2. Dynamic Latch Comparator

**Figure 13:**
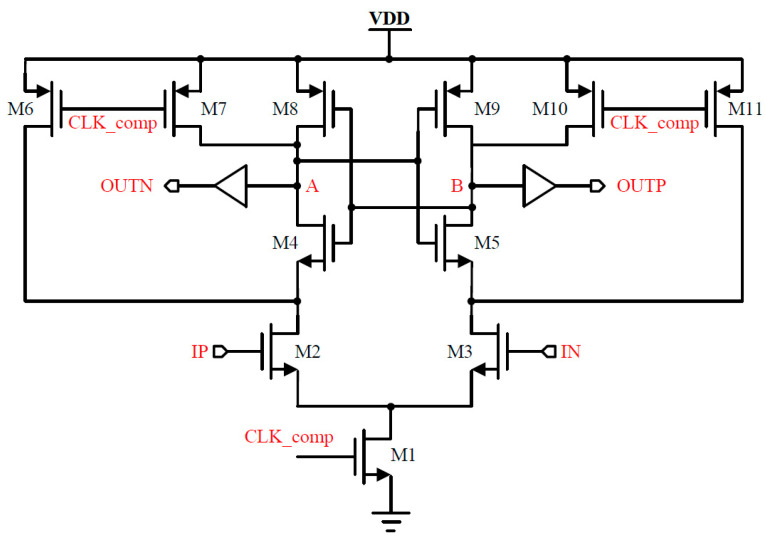
Circuit diagram of dynamic latch comparator.

**Figure 14:**
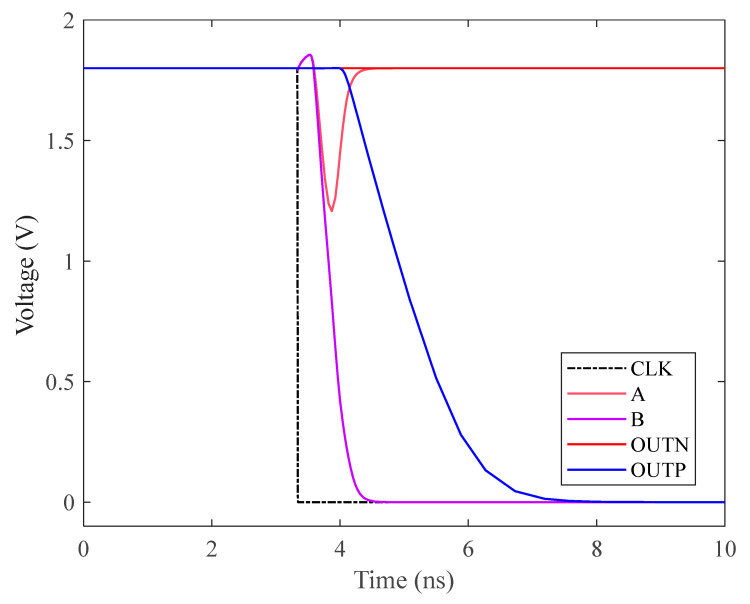
Transient simulation of dynamic comparator.

A clock-controlled, low-complexity, fully dynamic latched comparator with low power consumption is shown in Figure 13 [9,10]. The fully dynamic latched comparator uses NMOS as the input port to improve the comparison accuracy [11,12]. During the reset phase, A, B are reset to a high level. When the comparator starts working, the discharge rate of nodes A, B is related to the voltage of IP and IN. If IN > IP. The voltage of A is therefore greater than the voltage of B. Since M4, M5, M8 and M9 form a positive feedback latch circuit, eventually, the voltage of A will be higher, the voltage of B will be lower, OUTN will be higher and OUTP will be lower. If IP > IN, OUTP will become high, OUTN will become low, and the comparator completes the comparison. There is no DC path from VDD to ground during the operation of the comparator; therefore, only dynamic power is consumed. The transient simulation results of the comparator are shown in Figure 14.

#### 2.6.3. Dynamic SAR Controller

**Figure 15:**
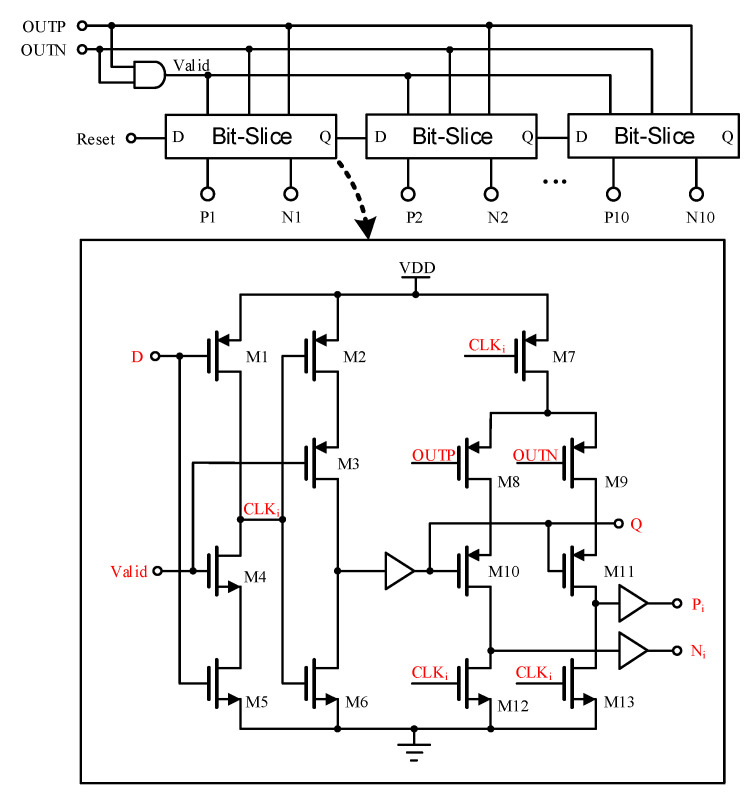
Dynamic SAR Controller.

Some studies [24,27,28,29] employ dynamic SAR logic, which significantly reduces the complexity of digital circuits and conserves a large number of transistors compared to conventional SAR logic. This leads to substantial reductions in power consumption and improved speed. In Figure 15, it is evident that the dynamic SAR logic comprises successive Bit-Slice circuits, each with the capability to shift and store comparison results.

When the reset signal becomes high to signal the end of the sampling phase, the D of the first dynamic logic becomes high, causing CLK1 to become low. At this juncture, if OUTP > OUTN, P1 becomes high and N1 remains low. When the Valid becomes low, P1 and N1 of the first comparison output are latched, and Q of the initial Bit-Slice circuit becomes high to indicate completion of the conversion. The subsequent Bit-Slice circuit repeats the operation of the first Bit-Slice circuit until all 10 bits of data have been stored.

## 3. Analysis of Results

The SAR ADC was designed and simulated utilizing 180 nm CMOS technology. The size of the unit capacitance C in the capacitor array is set to 4 μm × 4 μm, and C = 17.2 fF. According to the working principle of the logic analyzer, the logic analyzer function module is written in Verilog-A hardware description language for saving the simulation data.

**Figure 16:**
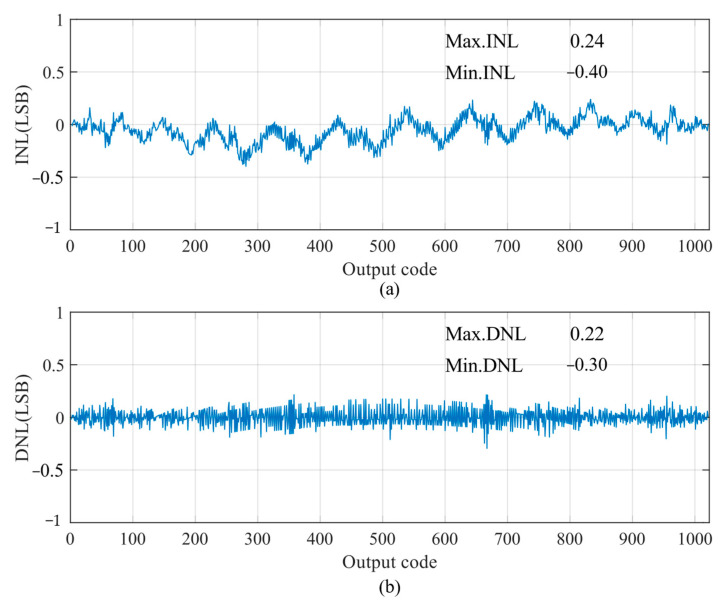
DNL and INL of proposed SAR ADC: (**a**) INL; and (**b**) DNL.

The static parameters are tested using the code density test (CDT) method [30]. The simulation parameters are as follows: the power supply is 1 V and the sampling rate is 100 kS/s. The simulated differential non-linearity (DNL) and integral non-linearity (INL) of the proposed SAR ADC are shown in Figure 16; the peak DNL and INL are −0.30 LSB ~ +0.22 LSB and −0.40 LSB ~ +0.24 LSB, respectively, which are less than 0.5 LSB, thus the designed circuit satisfies the static characterization requirement. The static performance of the proposed SAR ADC is somewhat limited by capacitor mismatch as a result of process gradient errors.

**Figure 17:**
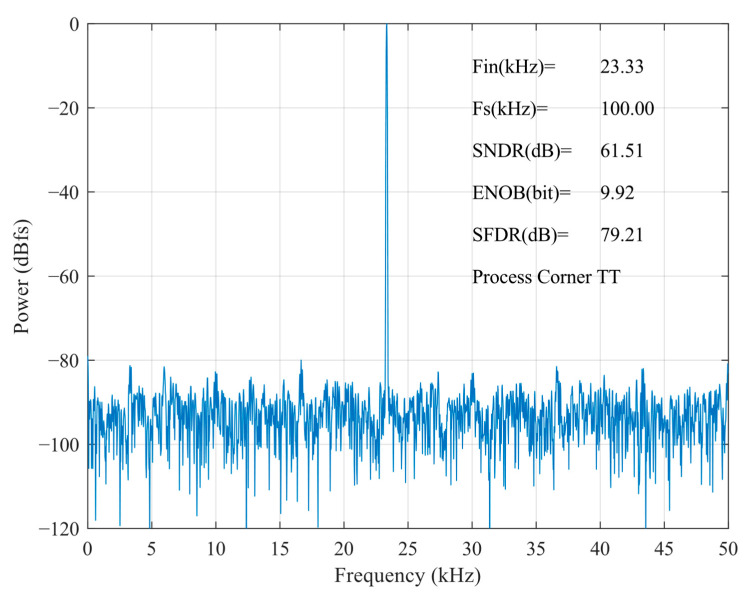
FFT of proposed SAR ADC at the TT process corner.

**Figure 18:**
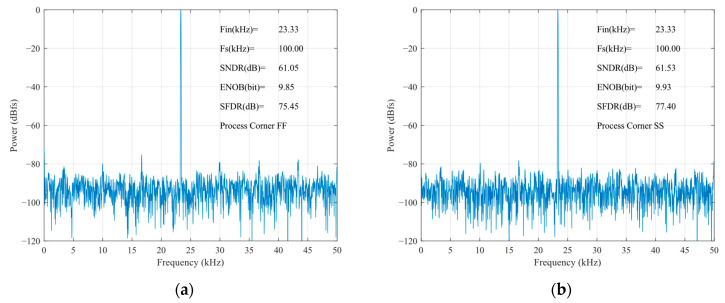
Process corner analysis: (**a**) FF process corner; and (**b**) SS process corner.

Figure 17 shows the FFT spectrum of the proposed SAR ADC; the harmonic components are not significant. The ADC achieves a 79.21 dB spurious-free dynamic range (SFDR), a 61.51 dB signal-to-noise and distortion ratio (SNDR), and the effective number of bits is 9.92 bits. The measured SFDRs demonstrate the good linearity of the proposed bootstrap S/H. The actual effective bits are affected by non-ideal factors, such as noise, misalignment, capacitance mismatch, etc., and the errors caused by these factors are within the acceptable range. Figure 18 shows the FFTs of the SAR ADC at different process corners. At the FF process corner, the ADC demonstrates an SNDR of 61.05 dB, an SFDR of 75.45 dB, and a 9.85 dB effective number of bits. At SS, the SNDR is 61.53 dB, the SFDR is 77.40 dB, and a 9.93 dB effective number of bits. Through the simulation and analysis of the process corners, it can be seen that the proposed scheme has a more stable performance under different process conditions, and the circuit has a certain degree of reliability.

**Figure 19:**
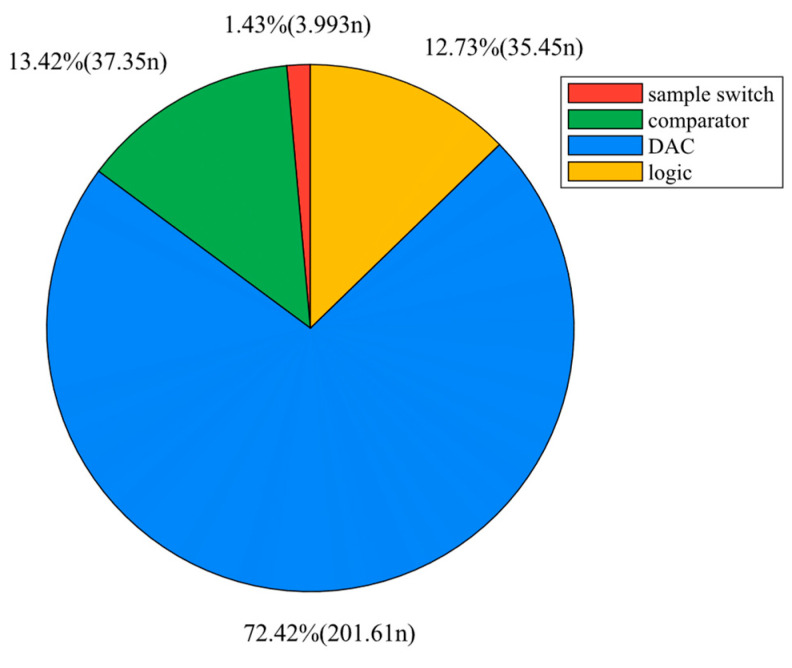
Power breakdown of proposed SAR ADC.

The average total power consumption of the SAR ADC is approximately 0.278 μW. The pie chart in Figure 19 displays the power consumption of each component, with the DAC and SAR dissipating the majority of the power. The comparison of the proposed ADC to other advanced SAR ADCs employs the figure of merit (*FOM*). According to [31], *FOM* is defined as follows:

(9) $$FOM=\frac{Power}{2^{ENOB}\times F_{s}}$$

where *F*_s_ is the sampling frequency, and *ENOB* is the effective number of bits of the Nyquist input, while Power is the total power consumption of the SAR ADC. The pro-posed SAR ADC achieves an *FOM* of 2.87 fJ/conv.-step, which is competitive.

The performance summary of some SAR ADCs [32,33,34,35], along with circuit-level simulation results of this work, are summarized in Table 6. The suggested ADC is clearly more efficient in terms of power usage and other metrics. It is highly energy-efficient, low in complexity, and small in area. Therefore, this ADC is a more advantageous choice in power- and area-constrained biosensor systems. The proposed ADC has many application possibilities since many biosensor devices need to detect analog signals. Moreover, performance can be further improved by using more modern CMOS technology.

## 4. Conclusions

This paper proposed a hybrid energy-efficient, area-efficient, low-complexity switching scheme in SAR ADC for biosensor applications. The proposed switching scheme saves 98.4% of the switching energy and reduces the number of unit capacitors by 87.5% compared to the conventional solution. At the loop bit of the conversion process, the scheme performs unilateral switching. Its voltage remains constant on one side. In addition, only six unit capacitors apply for *V*_aq_ as the reference voltage in the last two steps, and the remaining capacitors have only two reference voltages, which reduce the complexity of the circuit design. The drive circuit of this switching scheme has fewer logic gates than other schemes, meaning that it has low complexity. The advantages of the scheme are a low dependence on *V*_aq_, high energy efficiency, high area reduction, and low logic complexity. Furthermore, the simulation results demonstrated that the proposed SAR ADC achieves 61.51 dB SNDR, 79.21 dB SFDR and consumes 0.278 μW of power in a 180 nm process with a 1 V power supply, a full swing input signal frequency of 23.33 kHz, and a sampling rate of 100 kS/s.

## Figures and Tables

**Table 1:** Truth table for part of drive circuits I, J.

*P*_1_	*N*_1_	*P*_8_	*N*_8_	*P*_9_	*N*_9_	Reference Voltage of Unit C for High Array in Positive Array	Reference Voltage of Unit C for High Array in Negative Array
0	0	0	0	0	0	*V*_ref_	*V*_ref_
0	1	0	0	0	0	*V*_ref_	*gnd*
0	1	0	1	0	0	*V*_ref_	*gnd*
0	1	0	1	0	1	*V*_ref_	*gnd*
0	1	0	1	1	0	*V*_ref_	*V*_aq_
0	1	1	0	0	0	*V*_ref_	*gnd*
0	1	1	0	0	1	*V*_ref_	*gnd*
0	1	1	0	1	0	*V*_ref_	*V*_aq_
1	0	0	0	0	0	*gnd*	*V*_ref_
1	0	0	1	0	0	*gnd*	*V*_ref_
1	0	0	1	1	0	*gnd*	*V*_ref_
1	0	1	0	0	0	*gnd*	*V*_ref_
1	0	1	0	1	0	*gnd*	*V*_ref_
1	0	1	0	0	1	*V*_aq_	*V*_ref_
1	0	0	1	0	1	*V*_aq_	*V*_ref_

**Table 2:** Truth table for part of drive circuits K, L.

*P*_1_	*N*_1_	*P*_7_	*N*_7_	*P*_8_	*N*_8_	Reference Voltage of 2^N−10^ C(C) for Low Array in Positive Array	Reference Voltage of 2^N−10^ C(C) for Low Array in Negative Array
0	1	0	1	0	0	*V*_ref_	*gnd*
0	1	0	1	0	1	*V*_ref_	*V*_aq_
0	1	0	1	1	0	*V*_ref_	*V*_aq_
0	1	1	0	0	0	*gnd*	*gnd*
0	1	1	0	0	1	*gnd*	*V*_aq_
0	1	1	0	1	0	*gnd*	*V*_aq_
1	0	0	0	0	0	*gnd*	*gnd*
1	0	0	1	0	0	*gnd*	*gnd*
0	0	0	0	0	0	*gnd*	*gnd*
0	1	0	0	0	0	*gnd*	*gnd*
1	0	1	0	0	0	*gnd*	*V*_ref_
1	0	0	1	0	1	*V*_aq_	*gnd*
1	0	0	1	1	0	*V*_aq_	*gnd*
1	0	1	0	0	1	*V*_aq_	*V*_ref_
1	0	1	0	1	0	*V*_aq_	*V*_ref_

**Table 3:** Truth table for part of drive circuits M, N.

*P*_1_	*N*_1_	*P*_8_	*N*_8_	*P*_9_	*N*_9_	Reference Voltage of Unit C for Low Array in Positive Array	Reference Voltage of Unit C for Low Array in Negative Array
0	0	0	0	0	0	*gnd*	*gnd*
0	1	0	0	0	0	*gnd*	*gnd*
0	1	1	0	0	0	*gnd*	*V*_aq_
0	1	1	0	0	1	*gnd*	*gnd*
0	1	1	0	1	0	*gnd*	*V*_aq_
1	0	0	0	0	0	*gnd*	*gnd*
1	0	0	1	1	0	*gnd*	*gnd*
1	0	1	0	1	0	*gnd*	*V*_ref_
0	1	0	1	0	0	*V*_ref_	*V*_aq_
0	1	0	1	0	1	*V*_ref_	gnd
0	1	0	1	1	0	*V*_ref_	*V*_aq_
1	0	1	0	0	0	*V*_aq_	*V*_ref_
1	0	1	0	0	1	*V*_aq_	*V*_ref_
1	0	0	1	0	0	*V*_aq_	*gnd*
1	0	0	1	0	1	*V*_aq_	*gnd*

**Table 4:** Comparison of energy saving and area reduction for different switching schemes of a 10-bit SAR ADC.

Switching Scheme	Average Switching Energy (*CV*^2^_ref_)	Energy Saving	Area Reduction	Number of Unit Capacitors	Number of Switches for Each Capacitor
Conventional	1363.3	Reference	Reference	2048	2
ERMS [9]	128	90.61%	74.7%	518	2
Tri-level [10]	42.41	96.89%	75%	512	3
VMS [11]	31.88	97.7%	75%	512	3
Hybrid [12]	15.88	98.83%	75%	512	3
Wang et al. [13]	26.58	98.1%	87.5%	256	3/4 (only unit capacitor)
Li et al. [14]	26.67	98.0%	86.91%	268	3
Sanyal [15]	21.33	98.4%	75%	512	3
Wu et al. [16]	21.3	98.43%	75%	512	2
MAS [17]	21.1	98.45%	75%	512	2
Huang et al. [18]	5.3	99.61%	87.5%	256	3
Proposed	21.24	98.44%	87.5%	256	2/3 (only three unit capacitor)

**Table 5:** Comparison of dependency on the accuracy of *V*_cm_/*V*_aq_, logic complexity, common-mode variation and floating technique for different switching schemes of a 10-bit SAR ADC.

Switching Scheme	Logic Complexity	Dependency on the Accuracy of *V*_cm_/*V*_aq_	Common-Mode Voltage Variation	Floating Technich
Conventional	Low	No	0	No
ERMS [9]	Low	No	*V*_ref_/2	No
Tri-level [10]	High	Very high (all bits except MSB)	*V*_ref_/2	No
VMS [11]	High	Very high (all bits except MSB)	*V*_ref_/4	No
Hybrid [12]	High	Very high (all bits except MSB)	3*V*_ref_/8	No
Wang et al. [13]	Medium	High (all bits except MSB, LSB)	*V*_ref_/1024	No
Li et al. [14]	Medium	Very high (all bits except MSB)	*V*_ref_/4	No
Sanyal [15]	High	Very high (all bits except MSB)	*V*_ref_/4	No
Wu et al. [16]	Medium	No	*V*_ref_/2048	Yes
MAS [17]	Medium	No	*V*_ref_/1024	Yes
Huang et al. [18]	Low	High	3*V*_ref_/8	No
Proposed	Low	Only LSB and second LSB	*V*_ref_/4	No

**Table 6:** Performance comparison of proposed SAR ADC.

Parameter	[32]	[33] *	[34]	[35]	This Work *
Process (nm)	180	180	65	65	180
Resolution (bits)	8	10	11	10	10
Sampling Rate (MS/s)	30	0.001	0.1	3	0.1
Supply Voltage (V)	1.0/0.5	1	0.7	0.5	1
SNDR (dB)	46.3	60.3	59.4	54.6	61.51
ENOB (bits)	7.3	9.73	10.5	8.78	9.92
DNL (LSB)	−0.17/0.20	0.31	-	0.49	−0.30/0.22
INL (LSB)	−0.32/0.27	0.32	-	0.63	−0.40/0.24
Power Consumption (µW)	108	0.004	0.6	3.09	0.278
FOM (fJ/conv. Step)	22.8	4.8	4.5	2.34	2.87

* Simulated results.

## Data Availability

Data are contained within the article.
